# Feasibility and Preliminary Effects of the BESMILE-HF Program on Chronic Heart Failure Patients: A Pilot Randomized Controlled Trial

**DOI:** 10.3389/fcvm.2021.715207

**Published:** 2021-07-27

**Authors:** Xiankun Chen, Wei Jiang, Thomas P. Olson, Cecilia Stålsby Lundborg, Zehuai Wen, Weihui Lu, Gaetano Marrone

**Affiliations:** ^1^Health Systems and Policy, Department of Global Public Health, Karolinska Institutet, Stockholm, Sweden; ^2^Key Unit of Methodology in Clinical Research, Guangdong Provincial Hospital of Chinese Medicine, Guangzhou, China; ^3^The Second Affiliated Hospital of Guangzhou University of Chinese Medicine, Guangzhou, China; ^4^Department of Cardiology, Guangdong Provincial Hospital of Chinese Medicine, Guangzhou, China; ^5^Division of Cardiovascular Diseases, Department of Internal Medicine, Mayo Clinic and Foundation, Rochester, MN, United States; ^6^National Centre for Design Measurement and Evaluation in Clinical Research, Guangzhou University of Chinese Medicine, Guangzhou, China; ^7^Heart Failure Center/Department of Cardiology, Guangdong Provincial Hospital of Chinese Medicine, Guangzhou, China

**Keywords:** exercise-based cardiac rehabilitation, chronic heart failure, pilot randomized controlled trial, self-efficacy for exercise, *Baduanjin* exercise

## Abstract

**Aims:** The *Baduanjin* Eight-Silken-Movements wIth Self-Efficacy building for Heart Failure (BESMILE-HF) program is a contextually adapted cardiac rehabilitation program. It uses a traditional Chinese exercise, *Baduanjin*, to solve the unmet demand of exercise-based cardiac rehabilitation programs due to their scarcity and unaffordability in China. This pilot study assesses BESMILE-HF's feasibility and preliminary effects.

**Methods:** Eighteen patients with chronic heart failure were included: 8 in a BESMILE-HF group (age: 67 ± 5 years, EF: 40.4 ± 13.6%) and 10 in a control group (age: 70 ± 13 years, EF: 42.9 ± 12.5%). Both received the usual medications, with the intervention group receiving additionally the BESMILE-HF program for 6 weeks. Feasibility was explored by participants' involvement in the intended intervention. Clinical outcome assessments were conducted at baseline and post-intervention, while adverse events were captured throughout the study period.

**Results:** The BESMILE-HF program was well-received by patients, and adherence to the intervention was good. The intervention group completed all required home exercises and total home-practice time was correlated with baseline self-efficacy (*r* = 0.831, *p* = 0.011). Moreover, after 6 weeks, self-efficacy increased in the BESMILE-HF group (*p* = 0.028) and the change was higher than in the control [mean difference (MD): 3.2; 95% confidence interval (CI) 0.6–5.9, *p* = 0.004]. For the exercise capacity, the control group demonstrated a significant decline in peak oxygen consumption (*p* =0.018) whereas, the BESMILE-HF group maintained their exercise capacity (*p* = 0.063). Although the between-group difference was not statistically significance, there was clear clinical improvement in the BESMILE-HF group (1.5 mL/kg/min, 95% CI, −0.3 to 3.2 vs. minimal clinically important difference of 1 mL/kg/min). Throughout the study period, no adverse events related to the intervention were captured.

**Conclusions:** BESMILE-HF is feasible for patients with chronic heart failure in Chinese settings. A larger sample size and a longer follow-up period is needed to confirm its benefit on clinical outcomes.

**Clinical Trial Registration:**ClinicalTrials.gov: NCT03180320.

## Introduction

A hallmark symptom of chronic heart failure (CHF) is impaired exercise tolerance and poor quality of life. Exercise-based cardiac rehabilitation (EBCR) is a proven therapy to improve exercise capacity and quality of life in these patients (1, 2). However, the sub-optimal use of EBCR remains troubling and warrants high priority in global healthcare (3).

While many countries have reported gaps in patient referrals to existing EBCR programs, China has faced an even greater “upstream” challenge—a lack of available EBCR programs (4). One national survey showed that only 24% of China's tertiary hospitals have EBCR programs (5). Unfortunately, the type of comprehensive EBCR programs delivered in high income countries are not feasible in China due to the dearth of rehabilitation facilities, trained professionals, as well as unaffordability (5).

Home-based exercises can empower patients to take responsibility and accountability for their own disease management (6). Most importantly, they increase patients' access to EBCR by confronting the challenge of limited healthcare resources. This includes the paucity of rehabilitation facilities, the lack of medical reimbursement, and sub-standard access to hospital services in rural areas in China (7).

One possible solution tailored to the Chinese setting is traditional *Baduanjin* exercise which is usually practiced at home. *Baduanjin*, translated as Eight Silken Movements, is a form of ancient martial arts that originated in China and has been culturally accepted as being beneficial to one's health in Chinese society (8). This practice has evolved based on traditional Chinese medicine theory and is characterized by interplay between flowing circular physical postures and movements, mindfulness, and breathing exercise in harmony (9). *Baduanjin* is easy to learn and has minimal physical and cognitive demands because it entails only eight simple movements.

A novel and contextually adapted EBCR program using *Baduanjin*, BESMILE-HF, has recently been developed at the Guangdong Provincial Hospital of Chinese Medicine (GPHCM)—a tertiary care hospital and one of the oldest and largest Chinese medicine hospital groups in China (10). **BESMILE-HF** is an acronym for the **B**aduanjin **E**ight-**S**ilken-**M**ovement w**I**th Se**L**f-**E**fficacy building for **H**eart **F**ailure (10). In this program, *Baduanjin* has been applied as the core constituent in a multi-component EBCR including evaluation, consultancy, and education, as well as a series of self-efficacy building strategies to increase adherence, and to maintain exercise compliance over time.

However, uncertainties remain regarding the use of *Baduanjin* in an EBCR program. Therefore, we conducted a pilot study to: (1) assess the feasibility of the BESMILE-HF program regarding patients' adherence to their intended intervention protocols; and (2) attain initial estimates of the effects of the program on clinical outcomes.

## Methods

The study was conducted in accordance with the Declaration of Helsinki, and the BESMILE-HF study has been approved by the Ethics Committee at the GPHCM (number: B2016–202-01) and registered (ClinicalTrials.gov: NCT03180320). All patients were informed about the study, were given the possibility to ask questions and provided consent before participating in the study. Participants were told they could withdraw at any time.

### Design

This pilot study was a prospective, randomized controlled trial (RCT). This report includes the recommended elements elaborated upon in the reporting guidelines for pilot RCTs (Appendix 1: CONSORT checklist) (11).

### Setting

Guangzhou is the capital of Guangdong province and the 3rd-largest city in China. It is located in Southeastern China and has a permanent population of 13.5 million with over 7 million permanent residents in its urban areas (12). GPHCM is a tertiary care public hospital and has four branches in different urban districts of Guangzhou. In this hospital, cardiac rehabilitation is delivered one-on-one to individuals in the hospital outpatient clinic and includes exercise training. The most commonly used exercise is cycle ergometer. However, this service relies on out-of-pocket payment systems. This leads to a financial burden for most patients and results in low participation rate in cardiac rehabilitation.

### Participants

Recruitment took place at GPHCM from August to November 2017. Potential participants were identified for eligibility assessment by (1) on-site screening at clinic visits; (2) regular screening of potential participants using electronic medical records; and (3) referrals from physicians (10). Participants were included if they had clinically stable CHF with a New York Heart Association functional (NYHA) classification of II or III without restriction on left ventricular ejection fraction (LVEF) class. The complete list of inclusion and exclusion criteria has been reported previously (10) and is listed in Appendix 2.

### Schematic Process of the Pilot Study

**Figure 1:**
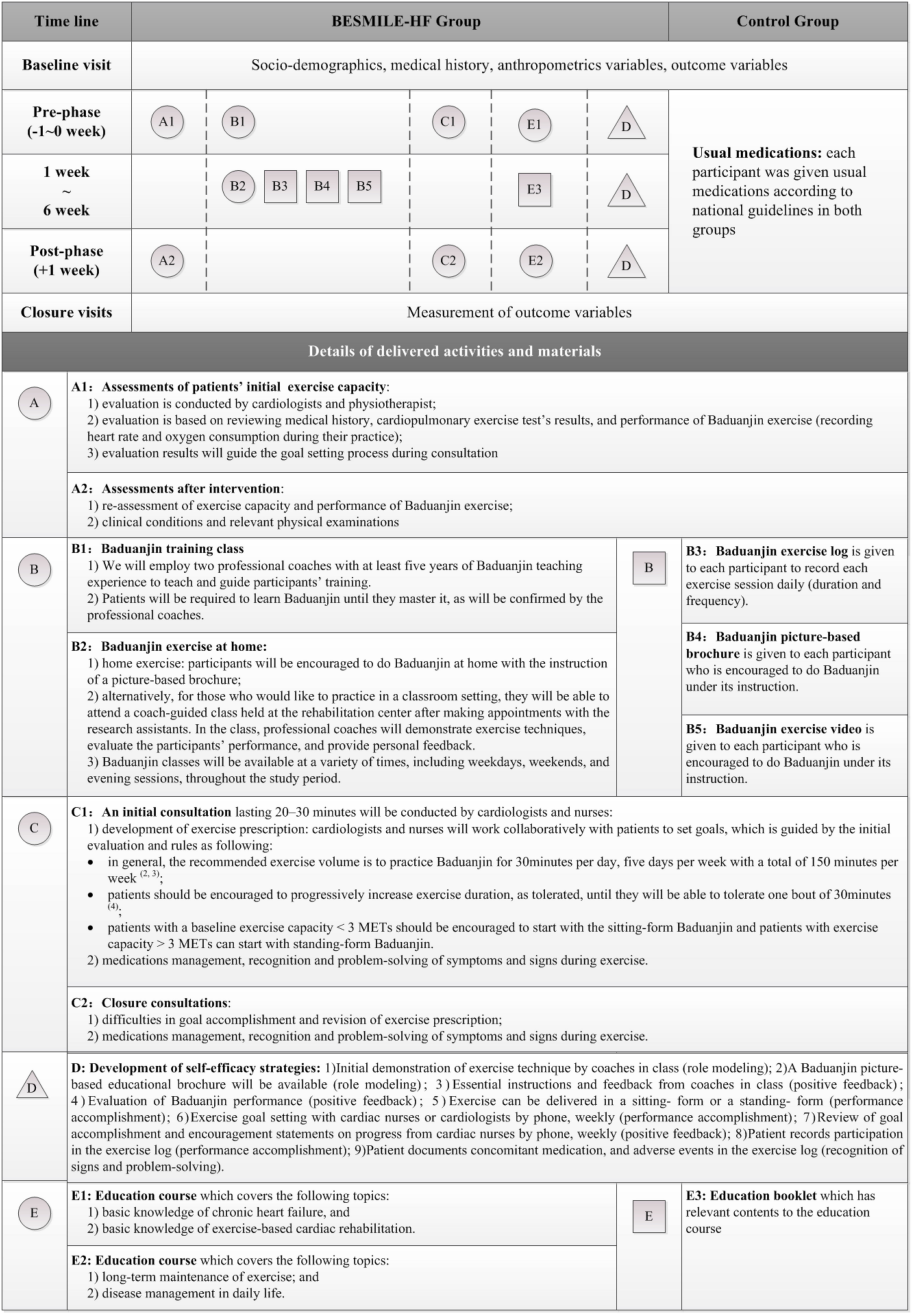
Graphical depiction of the BESMILE-HF program and the research schematic process for both groups, and data collection. Each component of the BESMILE-HF program is depicted separately. We regard components either as activities or materials planned to deliver to the patients. Activities are represented by circles (to reflect their flexibility) and materials by squares (to reflect their fixed nature). Different components are labeled with different letters. Below the diagram, a legend gives a brief description of each component.

The schematic process of the pilot study is shown in Figure 1. It is in accordance with the study protocol described previously (10).

#### Randomization, Allocation Concealment, and Blinding

Patients were informed and provided the possibility to ask questions before they signed a consent form. Eligible patients were randomized into either a BESMILE-HF group or a control group. A block randomization sequence was generated by SAS 9.2 (SAS Institute Inc., Cary, NC, USA) in a 1:1 ratio. In the pilot RCT, treatment allocation was conducted using sealed and numbered envelopes. We had made 20 numbered envelopes (a target sample size of 20 participants). As we were able to include 18 participants, hence only the first 18 envelopes were used. Given the nature of the intervention, it was not possible to blind the patients and personnel involved in conducting the programs. Outcome assessors, laboratory technicians, data managers, and statisticians were blinded to treatment allocations.

#### Intervention and Control

A graphical depiction of the intervention is shown in Figure 1 and the *Baduanjin* exercise video used in this study can be found online (13, 14). Both groups received the usual medications in accordance with national guidelines for 6 weeks (15). In the BESMILE-HF group, patients also received the pilot BESMILE-HF program. It included the core components of the full-scale 12-week BESMILE-HF program. Before the start of the 6-week home-exercise period, participants attended an exercise course to learn the eight postures at the hospital. A professional coach confirmed their performance. Following the exercise course, participants attended an educational course covering topics related to CHF, as well as exercise on the same day. Initial evaluation was conducted by the cardiologist by reviewing medical history, clinical examination results, and *Baduanjin* performance. Once the evaluation report was finalized, the initial consultation session was conducted by the cardiologist and the cardiology nurse. They would explain the exercise prescription and the results for the initial evaluation following pre-defined outlines. This was followed by 6-weeks of home exercise with guidance and instructions from a *Baduanjin* exercise demonstration video, a graphical exercise brochure, and weekly follow-up. Participants were generally required to do *Baduanjin* 30 min per day, 5 days per week, resulting in a total of 150 min per week. This was tailored according to individual evaluation results. Patients were asked to record their exercise performance in an exercise log (including duration in minutes and frequency) daily throughout the study period. After 6 weeks, participants were contacted to return to the hospital to attend the closure evaluation- and consultation-sessions.

### Data Collection

#### Baseline Data

The following baseline data were collected by questionnaires and through medical chart review: (1) socio-demographics; (2) medical history; and (3) anthropometric variables.

#### Patient Adherence to the Intervention

Exercise compliance was collected from the self-reported exercise log. This information, along with course attendance rate, assessment- and consultation-session attendance rate, allowed characterization of patients' adherence to the intervention.

#### Clinical Outcome Measures From Both Groups

The following clinical outcomes proposed for a future full-scale study were collected at baseline and follow-up at the 6th week during an assessment appointment at GPHCM's Heart Failure Center. We used a cardiopulmonary exercise test to measure exercise capacity; a timed up-and-go test to measure balance and mobility; echocardiography to measure cardiac function; as well as biomarkers including N-terminal B-type natriuretic peptide (NT-proBNP), high sensitive C-reactive protein (hsCRP), hemoglobin, and lipid profile. For psychological aspects, we measured quality of life using both a validated disease-specific questionnaire, the Minnesota Living with Heart Failure Questionnaire (MLHFQ) and a generic questionnaire, the EQ5D-visual analog scale; we used the Self-Efficacy for Exercise scale (SEE) to measure exercise self-efficacy; as well as the Hospital Anxiety and Depression Scale to measure depression and anxiety status. Clinical events, such as hospitalizations and major adverse cardiac events (MACEs) and safety outcomes (adverse events), were captured throughout the study period. Details of outcome measurements have been reported previously (10) and are listed in Appendix 3.

### Statistical Analysis

Baseline socio-demographic and clinical characteristics were summarized for both intervention and control groups. Continuous data were summarized as mean and deviation (SD), or as median and interquartile range (IQR); categorical data were summarized as counts and percentages. For outcome variables, a Wilcoxon signed-rank tests was used to examine changes from baseline to the 6th week within the groups. In addition, the analysis of the baseline, the 6th week, and change from baseline to the 6th week in the intervention group vs. control group was conducted using the Mann–Whitney *U* test. Moreover, Spearman correlation was used to explore the relationship between baseline self-efficacy and patients' total exercise time. Statistical analysis was performed in PASW Statistics 18.0 (IBM SPSS Inc., Armonk, New York, USA). *p* < 0.05 was considered statistically significant.

## Results

**Figure 2:**
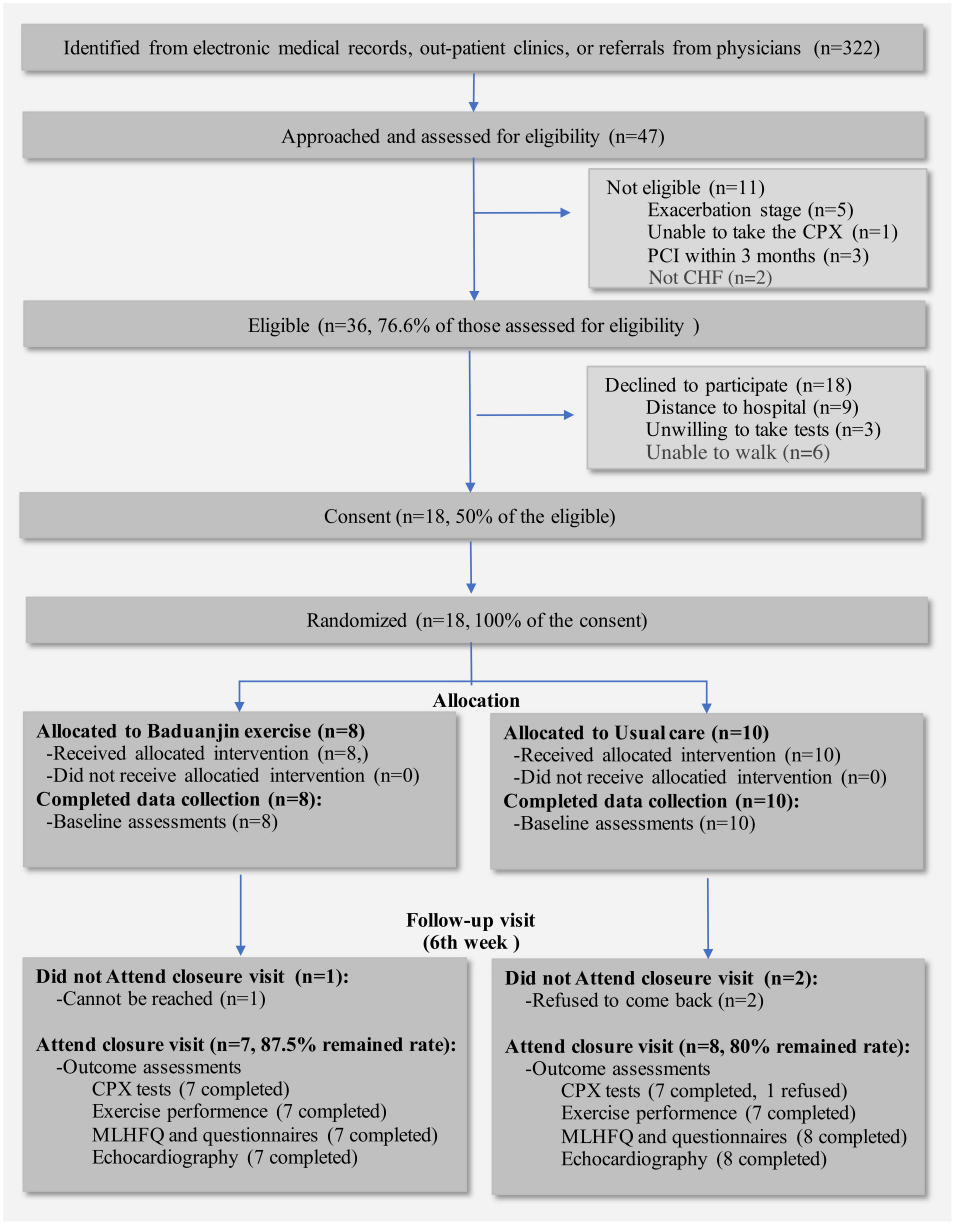
Flowchart of the pilot study. CPX, Cardiopulmonary exercise test; MLHFQ, Minnesota Living with Heart Failure Questionnaire.

A total of 322 individuals were identified. After exclusion of obvious exclusion criteria, such as contraindications to exercise or exercise test, not in the clinically stable stage, 47 (14.6%) were approached and screened for eligibility, and 36 (77%) met pre-defined criteria. They were all invited to participate in this pilot study and half of them (*n* = 18) refused to participate. Reasons were as followed: distance to the hospital (*n* = 9), unable to walk (*n* = 6), and unwilling to take the tests (*n* = 3). Female were more likely to refuse participations in our study (72.2% of the refused participants). Finally, a total of 18 (50% of those eligible) patients agreed to participate and were then randomized (Figure 2).

Baseline characteristics of the included participants are shown in Table 1. The majority of the participants were male (94%) with a mean age of 68 (SD: 10). Clinically, half were NYHA Class II, and the other half were NYHA Class III; 9 (50%), 4 (22%), and 5 (28%) patients had reduced, middle-ranged, and perceived LVEF, respectively. Beta-blockers were used by 89% of the participants. The intervention and control groups were comparable on all demographic and clinical characteristics, expect for history of coronary heart disease.

**Table 1:** Baseline characteristics of 18 participating patients.

	**All**	**Intervention**	**Control**
	***n*** **= 18**	***n*** **= 8**	***n*** **= 10**
**Demographics**			
Age, years	68 ± 10	67 ± 5	70 ± 13
Male, *n*	17 (94)	8 (100)	9 (90)
BMI, kg/m^2^	23 ± 3	23 ± 3	24 ± 3
Smoking			
Never smoke, *n*	9 (50)	5 (63)	4 (40)
Previous smoker, *n*	7 (39)	2 (25)	5 (50)
Current smoker, *n*	2 (11)	1 (13)	1 (10)
Marital status			
Married, *n*	17 (94)	8 (100)	9 (90)
Widowed, *n*	1 (6)	0 (0)	1 (10)
Education			
Primary or less, *n*	6 (33)	3 (38)	3 (30)
High school or above, *n*	12 (67)	5 (62)	7 (70)
**NYHA class**			
NYHAII, *n*	9 (50)	5 (63)	4 (40)
NYHAIII, *n*	9 (50)	3 (38)	6 (60)
**LVEF class**			
HFrEF (EF <40%), *n*	9 (50)	5 (63)	4 (40)
HFmrEF (EF 40–49%), *n*	4 (22)	0 (0)	4 (40)
HFpEF (EF ≥ 50), *n*	5 (28)	3 (38)	2 (20)
**Cardiac interventional procedure/surgical treatment**			
PCI, *n*	11 (61)	6 (75)	5 (50)
Pacemaker, *n*	4 (22)	1 (13)	3 (30)
ICD, *n*	1 (6)	0 (0)	1 (10)
CRT/CRT-D, *n*	1 (6)	0 (0)	1 (10)
Valvular surgery*, n*	1 (6)	1 (13)	0 (0)
Repairment of V-/A-septal	1 (6)	1 (13)	0 (0)
defect, *n*			
**Comorbidity**			
Average comorbidities per patient, mean	4 ± 2	5 ± 2	3 ± 1
Coronary heart diseases, *n*	13 (72)	8 (100)	5 (50)
Hypertension, *n*	11 (61)	6 (75)	5 (50)
Atrial fibrillation/flutter, *n*	6 (33)	3 (38)	3 (30)
Myocardial infarction, *n*	3 (17)	2 (25)	1 (10)
Stroke (Ischemic), *n*	2 (11)	2 (25)	0 (0)
Hyperlipoidemia, *n*	5 (28)	3 (38)	2 (20)
Type2-Diabetes, *n*	11 (61)	5 (63)	6 (60)
Hyperuricemia*, n*	11 (61)	5 (63)	6 (60)
COPD/Asthma*, n*	1 (6)	1 (13)	0 (0)
**Current cardiac relevant medications**			
ACEI/ARB, *n*	17 (94)	7 (88)	10 (100)
Beta-blockers, *n*	16 (89)	8 (100)	8 (80)
Aldosterone blockades, *n*	16 (89)	7 (88)	9 (90)
Statins, *n*	16 (89)	8 (100)	8 (80)
Platelet anti-aggregants, *n*	14 (78)	8 (100)	6 (60)
Diuretics, *n*	10 (56)	3 (38)	7 (70)
Digoxin, *n*	5 (28)	3 (38)	2 (20)
Calcium antagonists, *n*	4 (22)	1 (13)	3 (30)
Anticoagulant, *n*	3 (17)	0 (0)	3 (30)

*Results are presented as mean ± SD or n (%)*.

*BMI, Body Mass Index; NYHA, New York Heart Association; EF, ejection fraction; HFrEF, heart failure with reduced ejection fraction; HFmrEF, heart failure with mid-range of ejection fraction; HFpEF, heart failure with preserved ejection fraction; PCI, percutaneous coronary intervention; ICD, implantable cardioverter defibrillators; CRT/CRT-D, cardiac resynchronization therapy/with defibrillator; V-/A-, ventricular-or atrial-; COPD, chronic obstructive pulmonary disease; ACEI/ARB, angiotensin-converting enzyme inhibitors/angiotensin receptor blocker*.

### High Fidelity to Intervention

**Figure 3:**
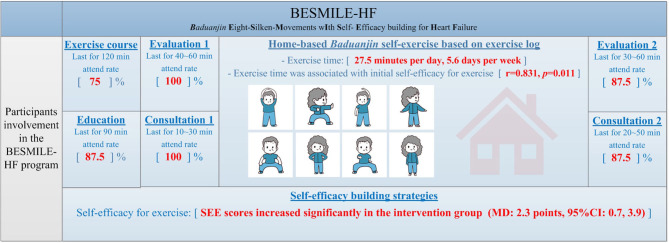
Participants involvement in the BESMILE-HF program (*n* = 8). Participants' involvement in the BESMILE-HF program regarding the self-report home exercise and attendant rates in other scheduled sessions are shown in red. The intervention group had done the required home exercises and total home-practice time was significantly related to baseline self-efficacy. SEE, Self-efficacy for exercise; MD, mean difference.

Participants involved in the BESMILE-HF program regarding the self-reported home exercise and attendant rates in other scheduled sessions are shown in Figure 3. Our results showed that the BESMILE-HF program was feasible with relatively high adherence. As a home-based EBCR program, the intervention group demonstrated good compliance with the required exercises. On average, participants exercised 27.5 (SD: 11.4) minutes/day and 5.6 (SD: 2.6) days/week for 6 weeks, reaching both the general required daily exercise time (30 min/day) and exercise frequency (5 days/week), respectively (Figure 3). Moreover, the total home-practice times (mins) had a significant positive relationship with their baseline self-efficacy scores (*r* = 0.831, *p* = 0.011).

In terms of other part of the BESMILE-HF program, all patients participated in the *Baduanjin* course which lasted for about 120 minutes and their performances were confirmed by the professional coach, except for 2 patients who preferred to learn the exercise via the video (attendance rate: 75%). All patients took the education course at baseline and at 6th week except one patient (overall attendance rate: 87.5%). All patients underwent baseline evaluation-session which included the collection of general assessments as well as individual cardiorespiratory data of *Baduanjin* exercise performance, except for two patients had only the general assessment data. All patients participated to the baseline consultation-session which mainly includes development of exercise prescription. For the closure evaluation- and consultation- sessions, all patients participated except one who died within 6-week (attendance rate: 87.5% for both sessions).

### Effects of the BESMILE-HF Program

In terms of the exercise capacity, the control group demonstrated a significant decline in VO_2peak_ (MD: −2.6, 95% CI −4.3 to −0.9), whereas, the BESMILE-HF group maintained their exercise capacity (MD: −1.2, 95% CI −1.2 to 0). Although the between-group difference was not statistically significance, there was a clear clinical improvement in the BESMILE-HF group (1.5 mL/kg/min, 95% CI: −0.3 to 3.2 vs. minimal clinically important difference of 1 mL/kg/min; Table 2.1).

**Table 2:** Clinical outcomes in each group and between-group comparison (*n* = 18).

**Outcomes**	**Control**		**Intervention**		***P*** **-value^e^**
	**Baseline**	**6th week**	**Baseline**	**6th week**	
**1. CPX parameters**					
Exercise test time, seconds	399 (309.8, 408.3)	329 (253, 419)	501 (400.8, 538.5)	404 (387, 488)	0.902
Wordload, watt	66 (53.3, 77.8)	70 (53, 85)	82.5 (65.8, 89.3)	82 (80, 85)	1
RER	55.5 (43.7, 59.5)	52.2 (40.3, 67.1)	60 (53.5, 68)	62.6 (62, 65.4)	1
VO_2at_, mL/kg/min^a^	1 (1, 1.1)	1.1 (1, 1.3)	1.1 (1, 1.2)	1.1 (1, 1.2)	0.165
VO_2peak_, mL/kg/min^a^	10.9 (8.9, 14.1)	9.7 (6.1, 11.6)^c^	12.9 (11, 13.6)	11 (10.3, 12.1)	0.128
VO_2peak_ %pred, %^a^	14.3 (10.2, 17.4)	13.6 (8.6, 15.2)^c^	15.9 (15.1, 16.8)	14.3 (13.6, 16.4)	0.295
HRR, bpm^a^	54.4 (49.4, 62.2)	47.6 (30.7, 51.7)^c^	59.2 (56, 62.6)	59.2 (50.6, 61.1)	0.128
Peak O_2_ pulse, mL/beat^a^	29.5 (14.5, 42.5)	25 (11, 34)	35 (25.3, 47)	26 (16, 40)	0.097
V_E_/VCO_2_, slope^b^	8.1 (7, 10)	7 (6.4, 9)^c^	8.9 (7.6, 10.7)	10 (7.7, 11.1)	0.805
dVO_2_/dWR, mL/min/W^a^	32.7 (29.1, 41.2)	36.7 (33.3, 42.9)^c^	32.9 (24.3, 35.9)	33.6 (28.2, 35.4)	0.073
FEV1 %pred, %^a^	7.4 (6.3, 8)	8.3 (4, 8.8)	7 (5.5, 8.7)	10.3 (9.4, 11.4)^d^	0.165
MVV %pred, %^a^	71 (60.8, 83.3)	68 (56, 87)	74 (54.5, 85)	78 (63, 82)	0.053
**2. Self-efficacy for exercise**					
SEE^a^	3.9 (3.1, 6.6)	4.3 (2.7, 5.1)	3.5 (1, 6.8)	7.2 (4, 9.9)^c^, ^d^	0.04
**3. Echocardiography**					
LVEF, %^a^	41 (35, 47.8)	36.5 (34, 39.5)	34.5 (30.5, 50)	45 (36, 53)	0.281
FS, %^a^	24 (19.5, 30)	19 (17, 22.5)	21 (17.3, 28.8)	27 (18, 29)^d^	0.04
SV, mL/bit^a^	80 (64, 83.5)	82.5 (63.8, 92.8)	66 (57.8, 86.8)	83 (70, 97)	0.165
LVEDD, mm^a^	60 (51.8, 63.5)	64 (59, 67.5)	60 (49.5, 70)	59 (51, 67)	0.281
PASP, mmHg^b^	31 (19, 45)	26 (20.3, 36.5)	31 (25.8, 43.8)	27 (25, 32)	0.536
**4. Biomarkers**					
NT-proBNP, pg/L^b^	1,177 (717.8, 2351)	872.9 (489.7, 4191.5)	1,346 (268.5, 2686.3)	703 (289, 1062)	0.397
hsCRP, mg/L^b^	5.4 (1.1, 10.8)	6.2 (1.8, 15.9)	2.4 (0.5, 5.7)	1.6 (1, 4.4)	0.694
Total cholesterol, mmol/L^b^	4.3 (3, 4.6)	3.7 (3.3, 4.2)	4.2 (3.5, 4.4)	4.1 (3.6, 4.7)	0.072
LDL-C, mmol/L^b^	2.4 (1.7, 3)	2.2 (1.7, 2.7)	2.4 (2.2, 2.9)	2.7 (1.9, 3)	0.336
HDL-C, mmol/L^a^	1.2 (0.8, 1.6)	1.1 (0.8, 1.6)	1 (1, 1.2)	1 (0.9, 1.3)	0.152
Triglycerides, mmol/L^b^	1.3 (0.6, 1.8)	1.1 (0.7, 1.5)	1.5 (1.1, 2.2)	1.2 (1, 1.7)	0.536
Hemoglobin, g/L^a^	130.5 (124.3, 141.8)	137 (127, 147)^c^	134 (125.5, 142)	135 (131, 142)	0.731
**5. Exercise performance**					
Timed-Up and Go, seconds^b^	8.1 (6.5, 10.8)	8.6 (7.8, 10.7)^c^	7.1 (6.6, 9.8)	7.7 (6.9, 8.7)	0.094
Left-hand grip strength, kg^a^	28.6 (20.8, 35.5)	28.7 (22, 37.3)	29.7 (23.5, 32.2)	31.2 (26.1, 33.1)	0.536
Right-hand grip strength, kg^a^	31.8 (22.9, 37.6)	27.6 (23.9, 35.3)	29.9 (26.4, 33.1)	27.4 (23.6, 27.5)^c^	0.397
**6. Quality of life**					
MLHFQ total score^b^	20.5 (13.8, 65.5)	16.5 (3.3, 23.5)	12.5 (5.5, 31.8)	12 (1, 26)	0.779
EQ-5D-VAS score^a^	80 (73.8, 90)	86.5 (80, 98.8)	85 (64.8, 93.8)	75 (70, 100)	0.232
**7. Depression and anxiety status**					
HADS-anxiety score^b^	1.5 (1, 7)	1 (1, 3)	0.5 (0, 3)	1 (0, 6)	0.867
HADS-depression score^b^	1 (1, 3)	2.5 (1, 5.5)	1 (0, 6)	0 (0, 1)	0.072

*Continuous data are summarized as median and interquartile range (IQR)*.

*CPX, cardiopulmonary exercise test; RER, respiratory exchange ratio; METs, metabolic equivalents; VO_2at_, oxygen uptake at AT; VO_2peak_, peak oxygen uptake; HRR, heart rate reserve 1 min post exercise; V_E_/VCO_2_, minute ventilation-carbon dioxide production; dVO_2_/dWR, the rate of increase in VO_2_ relative to work rate;FEV1 %pred, percentage of predicted value of forced expiratory volume in 1 min; MVV %pred, percentage of predicted value of maximum voluntary ventilation; LVEF, Left ventricular ejection fraction; FS, fractional shortening; SV, stroke volume; LVEDD, left ventricular end-diastolic diameter; PASP, pulmonary artery systolic pressure; NT-proBNP, N-terminal B-type natriuretic peptide; hsCRP, high-sensitivity C-reactive protein; LDL-C, low density lipoprotein-cholesterol; HDL-C, high density lipoprotein-cholesterol; MLHFQ, Minnesota Living with Heart Failure Questionnaire; EQ-5D-VAS, EQ-5D-visual analog scale; HADS, Hospital Anxiety and Depression Scale; SEE, Self-efficacy for exercise questionnaire*.

a *Higher value more favorable*.

b *Lower value more favorable*.

c *p < 0.05, refers to the comparison with baseline using Wilcoxon signed-rank tests*.

d *p < 0.05, refers to the comparison with the control group using Mann–Whitney U test at the 6th week*.

e *Comparison was conducted for the change from baseline to the 6th week in the intervention group vs. control group was conducted using the Mann–Whitney U test*.

After 6 weeks, SEE scores improved significantly in the BESMILE-HF group [mean difference (MD): 2.3, 95% confidence interval (CI) 0.7–3.9, *p* = 0.014; Table 2.2], but decreased slightly in the control group (MD: −0.9, 95% CI −3.2 to 1.4, *p* = 0.377; Table 2.2). When comparing the score changes between the two groups, a significant difference was found (MD: 3.2; 95% CI 0.6–5.9, *p* = 0.004).

For other clinical outcomes, no significant differences between groups, for either post-intervention values or changes, were observed for: most of the echocardiography parameters (Table 2.3); biomarkers such as NT-proBNP and hsCRP (Table 2.4); balance/mobility as measured by Timed up-and-go test (Table 2.5); quality of life (Table 2.6), or status of depression/anxiety (Table 2.7).

### Safety of the BESMILE-HF Program

Throughout the pilot RCT, no adverse events related to the intervention were captured. However, we documented several MACEs during the study period, one patient in the BESMILE-HF group died due to heart failure exacerbation, and two patients from the control group experienced acute heart failure exacerbation resulting in hospitalization.

## Discussion

The findings of this pilot study support the feasibility of the contextually adapted BESMILE-HF program using traditional *Baduanjin* exercise for patients with CHF in China. The BESMILE-HF program was well-received by patients. As a home-based EBCR program, the intervention group demonstrated exceptional compliance with the required exercises. We also found that one's initial self-efficacy had a positive effect on the total exercise time. More importantly, intervention can improve participants' exercise self-efficacy and may have benefit on exercise capacity.

The BESMILE-HF program's feasibility is primarily attributed to its in-home nature and the use of traditional *Baduanjin* exercise. According to a recent consensus statement on EBCR delivery in low-resource settings, safe, equipment-free, low-cost, and easy-to-implement exercise modalities provide the most practical options for Chinese settings (16). Compared to other low-cost outdoor activities such as walking, biking, running, and swimming, a home-based modality might be a more attractive and sensible option for optimal EBCR flexibility, given the fact that one-third of Chinese HF patients had difficulty or were unable to leave their homes due to their symptoms (17). In addition, *Baduanjin* exercise is an adaptable form of exercise that can be practiced in any place, and at any time. It also requires no special equipment and is not time-consuming. Hence, it is easy to be incorporated into daily routines.

Generally, adherence to exercise programs is low among CHF patients, which may limit its effect on clinical outcomes (18). In practice, self-efficacy plays a crucial role in adherence (19). In this study, a statistically significant positive relationship was found between baseline self-efficacy scores and individual exercise time. This result is supported by emerging literature in which self-efficacy is reported as the dominant factor in exercise uptake and maintenance among the CHF population (20). Therefore, it is reasonable to assume that the BESMILE-HF program might increase participants' adherence and maintenance of exercise compliance over time.

It is important to highlight that there was a significant improvement in self-efficacy score in the intervention group, but not in the control group. The between-group difference was found to be statistically significant, even within the context of this pilot study. However, the lack of periodic contact with doctors or nurses in the control group could have resulted in bias since the frequent contact with rehabilitation staff may explain some of the improvement in the intervention group. Nevertheless, a recent RCT reported that the 16-week *Baduanjin* training could improve self-efficacy for managing chronic diseases in community-living adults, such as increased confidence to mitigate fatigue, physical discomfort/pain, and emotional distress, and to be able to accomplish various tasks and activities (21). Our results also dovetail with previous evidence which shows that Tai Chi, a similar style of exercise, can improve CHF patients' self-efficacy (22).

Self-efficacy is defined as “the perceived confidence in the ability to take successful action and perform a specific task.” It is centered on four core elements: “role modeling,” “positive feedback,” “performance accomplishment,” and “recognition of problems and problem-solving” (23). In the BESMILE-HF program, specific adherence strategies for each of the four elements of self-efficacy were adopted and delivered as adjuncts to the *Baduanjin* exercise. Examples include an exercise course with an initial demonstration of exercise techniques by a coach and a graphic exercise brochure (role modeling); an evaluation session with feedback on *Baduanjin* performance and a weekly phone-call follow-up to review the progress by cardiac nurses (positive feedback); an exercise log to record individuals' own daily home-exercise (performance accomplishment); an educational course about disease management in daily life (recognition of problems and problem-solving).

The BESMILE-HF program is a complex intervention with several interacting components. This means that there will be a certain number of behaviors required by those delivering or receiving the intervention, as well as difficulties. Moreover, flexibility and tailoring of the intervention was permitted, as ensuring strict fidelity to a protocol may have been inappropriate. Evaluations of clinical outcomes are often undermined by problems such as delivery of the intervention, recruitment and retention, and smaller-than-expected effect sizes (24). Our small sample size was under-powered to reach a statistically significant effect on clinical outcomes such as VO_2peak_. However, there was a favorable trend with a clinically significant difference between two groups at the week 6 follow-up. In addition, our research team has recently confirmed that *Baduanjin* training intensity fulfilled ACSM's recommendations for bodily stimulation resulting in physiologically oriented outcomes (25). Moreover, previous studies have reported that *Baduanjin* improves exercise capacity (26–31). Therefore, the benefits of *Baduanjin* on exercise capacity should be expected from continued practice. Of note, *Baduanjin* can also be practiced in a sitting-form. Hece, for those patients with orthopedic limitations or other concomitant illness, the clinicians can also proposed this exercise to their patients to practice at home.

## Limitations

As with all studies, there are potential limitations to note. Firstly, the generalizability of the results might be limited by the characteristics of the included patients: NYHA II and III CHF patients who are relatively young and suffer from heart failure of mild-moderate severity. However, we still believe that our main findings described above are mostly applied to other parts of China. This is because the demographic and clinical characteristics of the study participants are similar to the those CHF patients undergoing a cross-sectional survey in Guangzhou (32) and in China as a whole (17). In addition, although we only recruited CHF patients with NYHA classification of II or III, the findings on *Baduanjin* intensity also apply to CHF patients in general. This is because patients with NYHA classification of II or III account for 83% of the HF patients in the stable stage in this setting (17). Secondly, 94% of the pilot study population was male. Sex has been showed to be an influencing factor in the change of VO_2peak_ and time spent on exercise (18, 33). Additional studies should strive to include an equal distribution of men and women. Thirdly, due to the nature of the intervention, blinding of patients and implementors was impossible. Trials with inadequate blinding are likely to exaggerate treatment effects, especially with regard to subjective results (such as SEE) and with participants with knowledge of traditional Chinese culture (34). However, we have blinded outcome assessors to minimize the detection bias. Fourthly, although the intervention group completed the required home exercises as reported through exercise logs, it should be noted that self-reported practice exercise tends to be overestimated (20). Assessing and ensuring adequate levels of intervention adherence is a challenge in most self-directed home-based interventions. However, self-reported exercise records, because of their ease of use, remain one of the most common tools for recording exercise data (20). Future, full-scale clinical trials should consider use of objective data collection methods to validate self-reported exercise data. Finally, given the scope of the pilot study and limited resources, the sample size was small. Therefore, the pilot RCT was not powered to test efficacy. However, the primary aim of the pilot study is to explore intervention feasibility, such as the recruitment rate, data collection process, and retention rate. Moreover, this small size study can still provide us some information regarding the preliminary efficacy of this intervention, as there is no data available regarding the BESMILE-HF program.

## Conclusion

This pilot study indicates that the BESMILE-HF program using traditional *Baduanjin* exercise, is feasible for patients with CHF in the Chinese setting. This practice may also increase patients' long-term adherence to exercise by improving exercise self-efficacy. Its potential benefits on clinical outcomes need confirmation with a larger sample size and a longer follow-up period. A full-scale RCT has been launched to determine the efficacy and safety of the BESMILE-HF program in patients with CHF.

## Data Availability Statement

The raw data supporting the conclusions of this article will be made available by the authors, without undue reservation.

## Ethics Statement

The studies involving human participants were reviewed and approved by Ethics Committee at the Guangdong Provincial Hospital of Chinese Medicine (number: B2016–202-01). The patients/participants provided their written informed consent to participate in this study.

## Author Contributions

XC, WJ, CL, ZW, WL, and GM contributed to the conception and design of the research. ZW and WL contributed to obtaining funding and supervising the work. XC drafted the first version of the manuscript and revised it based on other authors: WJ, TO, CL, ZW, WL, and GM contribution. All authors contributed important intellectual content to the critical revision of the manuscript and read and approved the final manuscript.

## Conflict of Interest

The authors declare that the research was conducted in the absence of any commercial or financial relationships that could be construed as a potential conflict of interest.

## Publisher's Note

All claims expressed in this article are solely those of the authors and do not necessarily represent those of their affiliated organizations, or those of the publisher, the editors and the reviewers. Any product that may be evaluated in this article, or claim that may be made by its manufacturer, is not guaranteed or endorsed by the publisher.

## Abbreviations

BESMILE-HF: Baduanjin Eight-Silken-Movement wIth SeLf-Efficacy building for Heart Failure
CHF: Chronic heart failure
CI: Confidence interval
EBCR: Exercise-based cardiac rehabilitation
GPHCM: Guangdong Provincial Hospital of Chinese Medicine
hsCRP: High sensity C-reactive protein
LVEF: Left ventricular ejection fraction
MACEs: Major adverse cardiac events
MD: Mean difference
MLHFQ: Minnesota Living with Heart Failure Questionnaire
NT-proBNP: N-terminal B-type natriuretic peptide
NYHA: New York Heart Association functional
RCT: Randomized controlled trial
SD: Standard deviation
SEE: Self-Efficacy for Exercise
VO_2peak_: Peak oxygen consumption.
