# Supplementary material for: Feasibility and Preliminary Effects of the BESMILE-HF Program on Chronic Heart Failure Patients: A Pilot Randomized Controlled Trial
Source: Front Cardiovasc Med. 2021 Jul 27;8:715207. doi: 10.3389/fcvm.2021.715207 (PMC8353081; doi:10.3389/fcvm.2021.715207)
Supplement: Supplementary file 1 [file Data_Sheet_1.docx]

**Appendix 1**

**
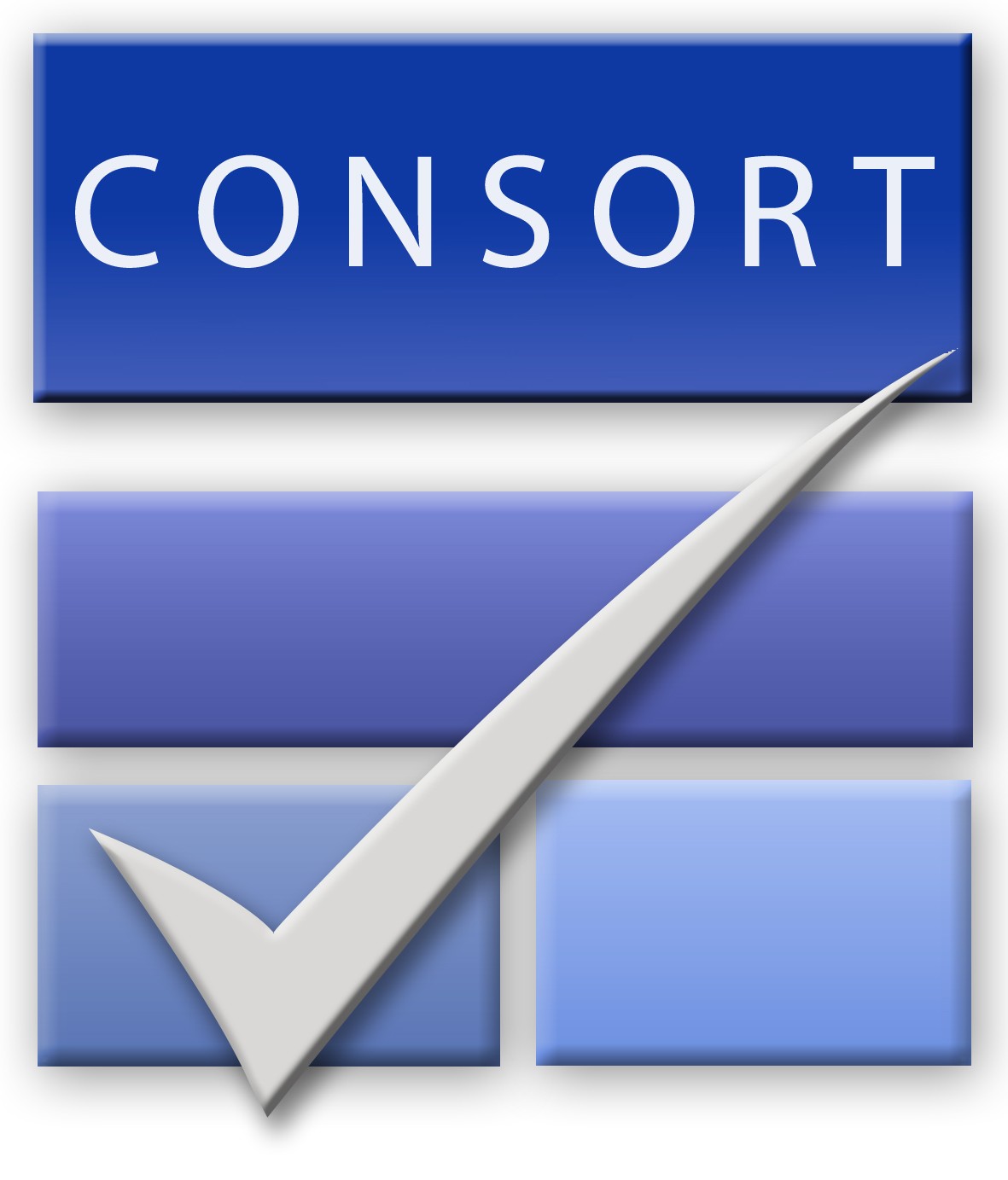
CONSORT 2010 checklist of information to include when reporting a pilot or feasibility trial***

| **Section/Topic** | **Item No** | **Checklist item** | **Reported on page No** |
| --- | --- | --- | --- |
| **Title and abstract** | | | |
|  | 1a | Identification as a pilot or feasibility randomised trial in the title | 1 |
|  | 1b | Structured summary of pilot trial design, methods, results, and conclusions (for specific guidance see CONSORT abstract extension for pilot trials) | 2-3 |
| **Introduction** | | | |
| Background and objectives | 2a | Scientific background and explanation of rationale for future definitive trial, and reasons for randomised pilot trial | 4-5 |
|  | 2b | Specific objectives or research questions for pilot trial | 5 |
| **Methods** | | | |
| Trial design | 3a | Description of pilot trial design (such as parallel, factorial) including allocation ratio | 5-7 |
|  | 3b | Important changes to methods after pilot trial commencement (such as eligibility criteria), with reasons | NA |
| Participants | 4a | Eligibility criteria for participants | 6 & Appendix2 |
|  | 4b | Settings and locations where the data were collected | 6 |
|  | 4c | How participants were identified and consented | 6-7 |
| Interventions | 5 | The interventions for each group with sufficient details to allow replication, including how and when they were actually administered | 7 & Figure1 |
| Outcomes | 6a | Completely defined prespecified assessments or measurements to address each pilot trial objective specified in 2b, including how and when they were assessed | 8 & Appendix3 |
|  | 6b | Any changes to pilot trial assessments or measurements after the pilot trial commenced, with reasons | NA |
|  | 6c | If applicable, prespecified criteria used to judge whether, or how, to proceed with future definitive trial | NA |
| Sample size | 7a | Rationale for numbers in the pilot trial | 9 |
|  | 7b | When applicable, explanation of any interim analyses and stopping guidelines | NA |
| Randomisation: |  |  |  |
| Sequence  generation | 8a | Method used to generate the random allocation sequence | 6 |
|  | 8b | Type of randomisation(s); details of any restriction (such as blocking and block size) | 6 |
| Allocation  concealment  mechanism | 9 | Mechanism used to implement the random allocation sequence (such as sequentially numbered containers), describing any steps taken to conceal the sequence until interventions were assigned | 6-7 |
| Implementation | 10 | Who generated the random allocation sequence, who enrolled participants, and who assigned participants to interventions | 6-7 |
| Blinding | 11a | If done, who was blinded after assignment to interventions (for example, participants, care providers, those assessing outcomes) and how | 6-7 |
|  | 11b | If relevant, description of the similarity of interventions | NA |
| Statistical methods | 12 | Methods used to address each pilot trial objective whether qualitative or quantitative | 9 |
| **Results** | | | |
| Participant flow (a diagram is strongly recommended) | 13a | For each group, the numbers of participants who were approached and/or assessed for eligibility, randomly assigned, received intended treatment, and were assessed for each objective | Figure 2 |
|  | 13b | For each group, losses and exclusions after randomisation, together with reasons | Figure 2 |
| Recruitment | 14a | Dates defining the periods of recruitment and follow-up | 6 |
|  | 14b | Why the pilot trial ended or was stopped | NA |
| Baseline data | 15 | A table showing baseline demographic and clinical characteristics for each group |  |
| Numbers analysed | 16 | For each objective, number of participants (denominator) included in each analysis. If relevant, these numbers should be by randomised group | 9 |
| Outcomes and estimation | 17 | For each objective, results including expressions of uncertainty (such as 95% confidence interval) for any  estimates. If relevant, these results should be by randomised group | Table 2 |
| Ancillary analyses | 18 | Results of any other analyses performed that could be used to inform the future definitive trial | NA |
| Harms | 19 | All important harms or unintended effects in each group (for specific guidance see CONSORT for harms) | NA |
|  | 19a | If relevant, other important unintended consequences | NA |
| **Discussion** | | | |
| Limitations | 20 | Pilot trial limitations, addressing sources of potential bias and remaining uncertainty about feasibility | 14 |
| Generalisability | 21 | Generalisability (applicability) of pilot trial methods and findings to future definitive trial and other studies | 14 |
| Interpretation | 22 | Interpretation consistent with pilot trial objectives and findings, balancing potential benefits and harms, and  considering other relevant evidence | 11-13 |
|  | 22a | Implications for progression from pilot to future definitive trial, including any proposed amendments | 14-15 |
| **Other information** | | |  |
| Registration | 23 | Registration number for pilot trial and name of trial registry | 5 |
| Protocol | 24 | Where the pilot trial protocol can be accessed, if available | Reference 11 |
| Funding | 25 | Sources of funding and other support (such as supply of drugs), role of funders | 16 |
|  | 26 | Ethical approval or approval by research review committee, confirmed with reference number | 5 |

Citation: Eldridge SM, Chan CL, Campbell MJ, Bond CM, Hopewell S, Thabane L, et al. CONSORT 2010 statement: extension to randomised pilot and feasibility trials. BMJ. 2016;355.

*We strongly recommend reading this statement in conjunction with the CONSORT 2010, extension to randomised pilot and feasibility trials, Explanation and Elaboration for important clarifications on all the items. If relevant, we also recommend reading CONSORT extensions for cluster randomised trials, non-inferiority and equivalence trials, non-pharmacological treatments, herbal interventions, and pragmatic trials. Additional extensions are forthcoming: for those and for up to date references relevant to this checklist, see [www.consort-statement.org](http://www.consort-statement.org).

**Appendix 2. BESMILE-HF pilot study inclusion and exclusion criteria**

| **Inclusion criteria** |
| --- |
| 1. aged 18 or above 2. diagnosed with chronic heart failure 3. clinically stable, defined as symptoms/signs that have remained generally unchanged for ≥1 month 4. New York Heart Association functional classification of II or III 5. Informed consent provided |
| **Exclusion criteria** |
| 1. patients who have contraindications to exercise testing: early phase after acute coronary syndrome (up to 6 weeks); life-threatening cardiac arrhythmias; acute heart failure (during the initial period of hemodynamic instability); uncontrolled hypertension (systolic blood pressure >200 mmHg and/or diastolic blood pressure >110 mmHg); advanced atrioventricular block; acute myocarditis and pericarditis; moderate to severe aortic/mitral stenosis; severe aortic/mitral regurgitation; severe hypertrophic obstructive cardiomyopathy; acute systemic illness; or intra cardiac thrombus. 2. patients who have contraindications to exercise training: progressive worsening of exercise tolerance or dyspnea at rest over the previous week; significant ischemia during low-intensity exercise (<2 Metabolic equivalents, <50 Watts); uncontrolled diabetes; recent embolism; thrombophlebitis; or new-onset atrial fibrillation/atrial flutter. 3. patients who have serious acute or chronic disease affecting major organs or mental disorders 4. history of cardiac surgery, cardiac resynchronization therapy, intracardiac defibrillation, or implantation of combined device within the previous 3 months 5. history of cardiac arrest within 1 year 6. history of peripartum cardiomyopathy, hyperthyroid heart disease, primary pulmonary hypertension 7. inability to perform a bicycle stress test 8. severe cognitive dysfunction precluding understanding of exercise concepts 9. current participation in either Baduanjin or a conventional cardiac rehabilitation program, 10. participation in a concurrent trial |

**Appendix 3：Details of outcome measurements**

Each participant was asked to attend an in-person assessment appointment at the Heart Failure Center of GPHCM at baseline and 6^th^. During each assessment, participants were asked to complete specified physiological tests and self-reported questionnaires. All tests were administered by the trained independent assessor.

**Cardiorespiratory fitness.** Cardiorespiratory fitness was assessed by measuring peak VO_2_ (mL/kg/min) according to guidelines.^(33)^ Participants performed a symptom-limited exercise test using a bicycle ramp protocol (10W/min) to determine peak VO_2_ (mL/kg/min). Testing was done on an electronically calibrated upright bicycle, with expired gas analysis under continuous electrocardiographic monitoring. Participants were encouraged to exercise until exhaustion. Perceived exertion was measured using the 6-20 Borg Scale. Technicians and physicians performing the tests were blinded to the study group. Oxygen uptake (VO_2_), minute ventilation-carbon dioxide production (VE/VCO_2_ slope), rate of increase in VO2 relative to work rate (∆VO2/∆WR), and O2 pulse were measured using a breath-by-breath respiratory gas analysis by the metabolic cart (Cardiovit CS-200 Touch, SCHILLER, Switzerland). These variables are reported to be particularly powerful in predicting prognosis of CHF patients. ^(33)^ Blood pressure were also taken at 3-minute intervals and just prior to starting/stopping exercise. The peak VO_2_ were defined as the highest value for or the plateau of oxygen uptake.

**Balance and mobility.** The timed up-and-go test (TUGT) was performed ^(34)^ and patients were required to stand up from a 45-centimeter high chair, walk 3 meters at a comfortable pace, turn 180°, return to the starting point, and sit again. This test was timed with the use of a standard stopwatch by a research assistant blinded to the treatment arm.

**Exercise self-efficacy.** Exercise self-efficacy was self-assessed by the Self-Efficacy for Exercise-Chinese scale (SEE-C). The participants was instructed to listen to nine different situations, and then to choose an option from 0 (not confident) to 10 (very confident) that represent their perception of confidence regarding engaging in regular exercise. The scale was scored by summing the numerical ratings for each response and dividing the total by the number of non-missing responses. The mean scores for the self-efficacy of exercise ranges from 0 to 10, with the higher scores representing greater exercise self-efficacy. A validated Chinese version of the SEE-C was employed in this trial.^(35)^

**Disease-specific quality of life.** The disease-specific quality of life was self-assessed by the MLHFQ. This validation instrument consists of 21 items rated on six-point Likert scales, representing different degrees of impact of CHF on quality of life, from 0 (none) to 5 (very much). It provides a total score (range 0–105, with a lower number denoting better quality of life), as well as scores for two dimensions, physical (8 items, range 0–40) and emotional (5 items, range 0–25). A validated Chinese version of the MLHFQ was employed in this trial. ^(36)^

**Generic quality of life.** Generic quality of life was self-assessed by the EQ-5D-5L questionnaire which has been used previously to measure health status in patients with heart failure.^(37, 38)^ In this pilot study, we only used the visual analog scale of EQ-5D-5L, a graph representation similar to a thermometer that ranges from 0 (worst imaginable health state) to 100 (best imaginable health state). It has been used previously to measure health status in patients with heart failure. A validated Chinese version of EQ-5D-5L was employed in this trial. ^(39)、^

**Anxiety and depressive status.** The Hospital Anxiety and Depression Scale (HADS) was self-administered to measure the presence of depression and anxiety. Participants were instructed to choose one response from the given answers that best describe their current feelings. The HADS is a 14-item self-report screening scale originally developed to indicate the possible presence of anxiety and depressive states in the setting of a medical out-patient clinic.

**Hallmark symptoms of dyspnea and fatigue*.*** Dyspnea and fatigue were self-assessed by the Modified Pulmonary Functional Status and Dyspnea Questionnaire (PFSDQ-M). The PFSDQ-M is comprised of 40 items divided into three components: dyspnea, fatigue, and activity. The activity domain evaluates changes in ten activities beginning at the point the patient first developed CHF: brushing/combing hair; putting on a shirt; washing hair; showering; raising arms overhead; preparing a snack; walking ten feet; walking on inclines; walking on bumpy terrain; and climbing 3 stairs. The dyspnea component evaluates dyspnea for frequency during the preceding month; intensity of dyspnea on most days, the present day, and with usual activity levels (rated on a scale from 0 to 10); and intensity of dyspnea when the above-mentioned ten activities are performed (rated on a scale from 0 to 10). The fatigue component evaluates fatigue for the same items as the dyspnea component. Higher scores indicate more severe symptoms. The PFSDQ-M is validated and is reliable in patients with CHF. A validated Chinese version was employed in this trial.^(40)^

**Echocardiography**. Echocardiography parameters of cardiac function was assessed by the resting three-dimensional multi-view echocardiogram before the exercise program was initiated and upon completion of the program. Functional parametes such as left ventricular ejection fraction (LVEF) as well as structural parameters related to diastolic/systolic/valvular status were also be collected.

**Laboratory tests.** Blood samples was collected to measure the levels of prognostic biomarkers (NT-proBNP) and inflammatory mediators (hsCRP). NT-proBNP and hsCRP were analyzed by the GPHCM lab. The NT-proBNP is correlated with risk for all-cause, cardiac, and pump-failure mortality, and hsCRP is elevated in CHF as the disease progresses.^(41)^ In addition, the following tests were also conducted using the blood sample: metabolic profile such as plasma levels of TG (mmol/L), TC (mmol/L), HDL-C (mmol/L), and LDL-C (mmol/L); Cr (mmol/L), Glu (mmol/L); Hb (g/L); plasma levels of K^+^ (mmol/L), Na^+^(mmol/L), and Cl^-^(mmol/L).

**Major adverse cardiac events (MACEs).** MACE components in our study included events of death, myocardial infarction, stent thrombosis, percutaneous coronary interventions, coronary artery bypass graft surgery, and stroke.

**Hospitalization related outcomes.** All instances of hospitalization were recorded throughout the study period and made accessible to an independent adjudication panel consisting of three experienced cardiologists. They were ascertain whether or not the reported events are heart failure-related.

**Medication usage.** Usual medical management as well as concomitant medications were documented in detail by cardiologists. In addition, concomitant medications was recorded in participants' exercise logs. Medical records, interview data, and automated utilization data were also be used to document patients’ use of healthcare throughout the study period.
